# Artificial Intelligence in Infectious Disease Care: Selected Applications in Tuberculosis, Sepsis, and Antimicrobial Stewardship

**DOI:** 10.3390/diagnostics16121827

**Published:** 2026-06-12

**Authors:** Olga Adriana Caliman-Sturdza, Roxana Elena Gheorghita, Roxana Filip, Andrei Lobiuc

**Affiliations:** 1 Faculty of Medicine and Biological Sciences, Stefan cel Mare University of Suceava, 720229 Suceava, Romania; olga.caliman-sturdza@usm.ro (O.A.C.-S.); andrei.lobiuc@usm.ro (A.L.); 2 “Sfântul Ioan cel Nou” Emergency Clinical Hospital, 720262 Suceava, Romania

**Keywords:** deep learning, machine learning, infection, diagnosis accuracy, treatment monitoring

## Abstract

**Background/Objectives**: Artificial intelligence (AI) is increasingly being applied across the infectious-disease pathway, from syndromic surveillance and imaging triage to etiologic support, antimicrobial stewardship, and prognostication. However, the maturity of evidence differs considerably across use cases, and apparent technical performance does not always translate into real-world clinical utility. **Methods**: This structured narrative review synthesizes current evidence on the principal clinical and public-health applications of AI in infectious diseases, with particular attention to external validation, workflow integration, economic implications, and governance. **Results**: The strongest near-term evidence supports narrow-AI applications linked to constrained workflows, especially tuberculosis chest-radiograph triage, selected host-response and antimicrobial-resistance prediction tools, and clinician-facing stewardship aids. By contrast, sepsis prediction illustrates how internal model performance may deteriorate on external validation and generate substantial alert burden when implemented in routine care. Economic evaluations are promising but remain predominantly model-based and context-dependent. Evidence for generative AI and large language models is still in an early phase, consisting largely of vignette studies, retrospective comparisons, and small single-center pilots rather than prospective outcome-based evaluations. **Conclusions**: Overall, the most realistic clinical role of AI in infectious diseases is augmentation rather than replacement: prioritizing scarce diagnostic capacity, shortening time to action, and improving antibiotic selection. Safe translation into practice requires, in order, external validation with local calibration, prospective impact assessment, and governance frameworks that address drift, accountability, transparency, and human oversight.

## 1. Introduction

Artificial intelligence (AI) has now been integrated throughout the infectious-disease pathway: at the early stage (case detection—triage and surveillance) to etiologic diagnosis, antimicrobial selection, dose advice, and prognostication [1,2]. The oldest clinical applications are so-called narrow AI systems: deep learning image triage for high-burden diseases such as tuberculosis (TB) and risk stratification/decision support for sepsis-like syndromes [3]. The quality of evidence is on the rise, and the general situation remains in the retrospective assessment and workflow-oriented implementation research instead of patient-centered randomized trials [4]. The most probable locations where AI will do better than normal care include conditions with constraints in the number of specialists available, the quantity of cases, and decision-making time (e.g., sepsis escalation, radiograph screening of TB, empiric antibiotic matching) [5,6,7]. Nonetheless, the identical circumstances increase the damage through models moving out of distribution (new pathogens, new test platforms, new admission patterns, and alterations in the ecology of antibiotic resistance). One of the most important lessons of real-world infectious-disease machine learning (ML) is that performance may decrease significantly on external validation, even when the tool is used widely [8]. The clinical utility of AI is maximum near-term value as an augmentation, not replacement: faster time to action (earlier isolation, earlier appropriate antibiotics), scarce diagnostic capacity prioritization (radiology, microbiology), and less inappropriate exposure to antibiotics with individualized forecasts of resistance and guideline-conscious sets of orders [9,10]. Critical limitations persist in stringent prospective impact assessments, calibration and monitoring in dataset/epidemiologic drift, governance and privacy-by-design integration which do not lead to alert fatigue and still maintain clinician accountability [11,12]. The present review therefore focuses less on headline model performance and more on clinical readiness. Specifically, we synthesize evidence across diagnostic triage, antimicrobial decision support, workflow integration, economic evaluation, and generative-AI applications in infectious diseases. Our aim is to distinguish comparatively mature, workflow-linked use cases from those that remain exploratory, and to identify the conditions under which AI may improve care without weakening clinician accountability.

## 2. Materials and Methods

This article was conducted as a structured narrative review intended to synthesize clinically relevant applications of artificial intelligence in infectious diseases rather than to provide an exhaustive systematic inventory of all published studies. To identify relevant literature, we searched PubMed, Web of Science, Scopus, and Embase for studies published from January 2021 through March 2026. The search strategy combined terms related to AI methods and infectious-disease applications, including artificial intelligence, machine learning, deep learning, natural language processing, infection, diagnosis, treatment, tuberculosis, image interpretation, laboratory interpretation, sepsis prediction, antimicrobial resistance, and cost-effectiveness. These terms were used in various combinations using Boolean operators “AND” and “OR”. Citation chaining and manual searching were used to identify additional influential or implementation-relevant papers. Selected official documents from regulators and international organizations were also consulted when directly relevant to implementation, governance, or regulatory status.

Sources were reviewed iteratively by two authors to improve consistency of selection and interpretation. Priority was given to studies that were clinically informative, methodologically influential, externally validated, prospectively evaluated, randomized or pragmatic in design, or especially relevant to implementation, economics, stewardship, or governance. We also retained a limited number of landmark older studies when they were necessary for historical framing.

We excluded publications that were not focused on infectious diseases, were unrelated to clinical or public-health uses of AI, were conference abstracts without sufficient detail, or addressed administrative applications without clear analytical relevance to infectious-disease care. Because the purpose of the review was interpretive thematic synthesis, not formal systematic inclusion, we do not present article counts as systematic-review outputs and do not provide a PRISMA flow diagram. Instead, evidence is synthesized thematically across diagnostic and triage tools, treatment support, workflow integration and validation, economic evaluation, and generative-AI applications.

## 3. Definitions and Scope of AI in Infectious Disease

AI in healthcare refers to algorithmic systems that perform tasks normally requiring human intelligence (e.g., recognition, prediction, decision support). One of the most recent developments is generative AI which includes large multimodal models capable of taking in various types of inputs and producing outputs such as text summaries and recommendations [13].

**Figure 1:**
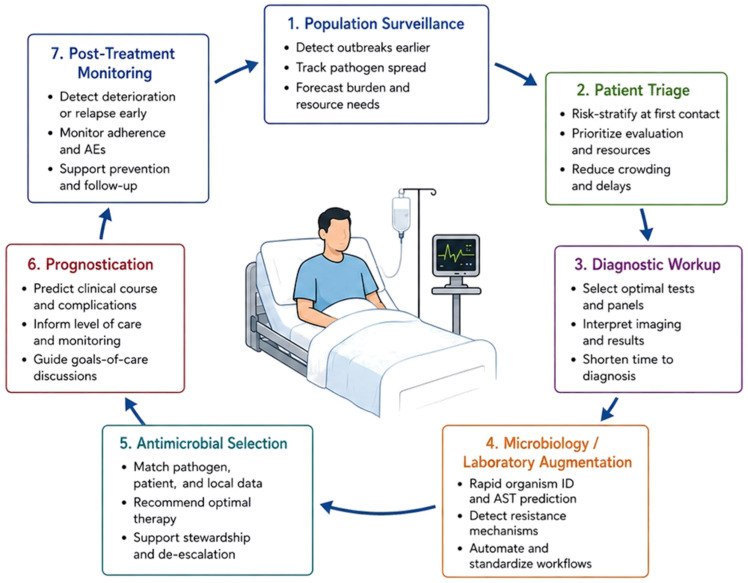
Workflow indicating where AI enters infectious disease care, moving from population surveillance and patient triage through diagnostic workup, microbiology/laboratory augmentation, antimicrobial selection, prognostication, and post-treatment monitoring.

In infectious diseases, AI applications are available across four levels of operation, including patient-level diagnosis and risk forecasting, microbiology/lab interpretation and rapid phenotyping, hospital/unit operations (antimicrobial stewardship, facility-level outbreak detection), and population-level syndromic surveillance and forecasting. The most common example of clinical framing is that AI tools can intervene at various stages of the infection management process, such as diagnosis, antimicrobial selection, and escalation decisions, but that they are constrained by the quality of their data, applicability, and rigor of their assessments [14] (Figure 1).

**Figure 2:**
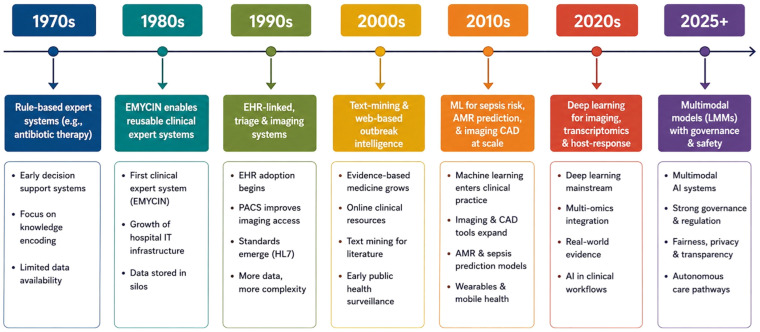
Evolution of clinically relevant AI applications in infectious diseases, organized by diagnostic triage, treatment support, surveillance, and generative-AI functions. The figure emphasizes evidence maturity rather than chronology alone. ML (machine learning), AMR (antimicrobial resistance), CAD (computer-aided diagnosis), LMMs (large multimodal models), and EHR (electronic health record).

There are various methods that are the core AI method families applicable in infectious diseases. Expert systems (rule-based) are knowledge bases and inference engines that encode clinical rules (“if–then”) for diagnosis and therapy selection. Infectious diseases are historically central here—MYCIN was explicitly designed for bacterial infection diagnosis and antibiotic therapy selection using production rules and explanation functions [15]. ML are statistical learning from labeled data (supervised) or structure discovery (unsupervised). In infectious diseases, ML often targets resistance prediction, sepsis risk, or test interpretation from routine electronic health record (EHR) variables [16]. Deep learning (DL) systems are neural networks (e.g., Convolutional neural networks—CNNs with imaging; sequence models with time-series EHR) adapted to high-dimensional data, i.e., chest radiographs, microscopy images, waveform/vitals streams, and multimodal input [17]. The process of extracting information in unstructured text (clinical notes, radiology reports, microbiology comments, public reports/news) are referred to as natural language processing (NLP) [18]. Transformer models are also part of modern NLP and can be applied to digital surveillance, as well as synthesize clinical documentation, although they need strictly confined use in safety-critical decision-making [19] (Figure 2).

## 4. Current Clinical Applications in Diagnosis and Treatment

The clinical diagnostic AI in infectious disease falls into four high-yield categories: imaging interpretation, lab test interpretation and rapid phenotyping, syndromic monitoring, and rapid/host-response diagnostics [20]. The most established imaging-based diagnosis and triage in TB screening/triage is due to the extensive use of chest radiography and limited capacity of expert radiology [21]. In a major study of five commercial algorithms of TB triage on 23,954 chest X-rays, all algorithms outperformed radiologists significantly; their areas under the curve (AUCs) reported to be 0.85–0.91 based on the product, and were compared to a target product profile of triage tests [22]. The BMC Infectious Diseases paper of Luo et al. is best used as a proof-of-concept TB differential-diagnosis study [23]. It enrolled 892 participants in a discovery cohort and 263 in a validation cohort; the best conditional random forest model achieved an AUC of 0.978 in the test set and 0.963 in the validation set, with validation sensitivity of 92.80% and specificity of 89.86% [23]. These are strong discrimination numbers, but the paper still represents a relatively early diagnostic modeling stage rather than real-world clinical implementation. In another study on the role of machine learning in discriminating between active and latent TB published by Luo et al. in *Journal of Infection*, 2619 participants formed the discovery cohort and 942 the independent validation cohort. The gradient boosting model had test-set sensitivity of 84.38% and specificity of 92.71%, with validation sensitivity of 87.63% and specificity of 91.34% [24]. A South African external validation compared 12 commercial TB CAD products and found substantial heterogeneity in AUC and threshold behavior; Lunit and Nexus had AUCs near 0.9, some products-maintained sensitivity above 90% over wide threshold ranges, and performance was worse in older adults, people with prior TB, and those with HIV [25]. The 2025 AIRIS-TB paper reports temporal, quasi-prospective evaluation on more than one million chest radiographs, with AUC 98.51%, overall false-negative rate 1.57%, and 0% TB-specific false-negative rate after expert review correction; it also includes subgroup analyses and an explicit workflow argument about replacing review of highly likely normal studies [26].

Lab-based diagnostic artificial intelligence is becoming more common to reduce the time interval between specimen and action. A key trend here is the ability to forecast antimicrobial resistance (AMR) based on routinely available microbiology indicators, e.g., Matrix-Assisted Laser Desorption/Ionization—Time of Flight (MALDI-TOF) mass spectra, without having to wait until culture-based susceptibility tests are performed [27]. This is to squeeze time to appropriate therapy, in environments where traditional workflows may take up to 72 h to complete finalized antimicrobial susceptibility testing (AST) [28]. Host-response/rapid diagnostics is another expansion: instead of detecting a pathogen, they assume that the immunological reaction of the patient is more typical of bacterial vs. viral infection and determine the risk of severity. The AUCs reported in a prospective study of emergency-department cohort study that validated a 29-mRNA host response classifier were 0.76 (bacterial) and 0.89 (viral), and a severity classifier AUC of 0.77 for 30-day mortality [29]. The study by Liu et al. published in 2025 is a large retrospective sepsis triage model, which aimed to develop and validate a sepsis prediction model [30]. Using structured triage data from 189,617 patients, the best gradient boosting model achieved an AUC of 0.83, and the paper also reported sensitivity of 0.74, specificity of 0.78, PPV of 0.77, and NPV of 0.74 for its best model 2 configuration [30]. Crucially, the study itself notes that further prospective and diverse-cohort validation is still required before real-world deployment. Syndromic surveillance AI, frequently NLP-based, is an open-source (news, social content) processing system that may use epidemiologic and environmental signals to issue earlier warnings of outbreaks [31]. It has been stressed in reviews that these systems should be regarded as supplements to the conventional surveillance and not as replacements, and that they must be carefully filtered and validated to minimize false alarms and amplification of misinformation [19].

Empirical antimicrobial selection (including resistance prediction), dosing optimization, prognosis/level-of-care decision-making, and clinician-facing decision support is commonly a target of AI in treatment decisions in infectious diseases [32,33]. There are signs that AI-based decision support can have an impact on antibiotic choice and, in other settings, outcomes. In a randomized controlled trial of *Stenotrophomonas maltophilia* infections, an AI clinical decision support system (AI-CDSS) based on MALDI-TOF predictions on infection yielded 1-day earlier resistance prediction (leading to real patient benefit: 14-day mortality in the AI arm was reduced compared to the control arm, 11.5 vs. 15.1, *p* = 0.03) and more optimal antibiotic decisions [34].

Guideline-conscious order sets with personalized resistance projections are used in outpatient care to minimize the inappropriate use of empiric therapy and the wasteful use of broad-spectrum antibiotics. A big real-world study of an “UTI Smart-Set” decision-support tool found it had lower rates of mismatch with recommendations taken (example figures reported include 8.9% vs. 14.2%), and also a massive decrease in ciprofloxacin use (example figures reported include 6.4% vs. 32.9%), with tool uptake being high [35]. For antimicrobial stewardship, the 2025 UTI Smart-Set implementation study is especially valuable because it moves beyond diagnostic performance into real behavior change. The optimization of dosing is also commonly described as model informed precision dosing, especially when dealing with antimicrobials with narrow therapeutic indices (e.g., vancomycin). Guidelines on professional consensus propose AUC-directed monitoring goals (AUC/MIC 400 or 600 under particular conditions), which generates the need to look for software capable of approximating exposure and recommending individual dose changes [36]. Machine learning analysis of the global microbiome has identified nearly one million potential antimicrobial peptides (AMPs), offering a significant resource for combating antibiotic resistance. The study by Santos-Júnior published in *Cell*, found that 79 synthesized candidate peptides effectively disrupted bacterial membranes, with leading candidates showing preclinical success comparable to current antibiotics [37]. That is exciting translational science, but it is not yet a bedside stewardship tool. The external validation of the Epic Sepsis Model is one of the clearest demonstrations of why external validation matters: hospitalization-level AUC was 0.63, and at a threshold of six the model had sensitivity 33%, specificity 83%, PPV 12%, and NPV 95% [8] (Table S1). At the same time, a 2024 meta-analysis of sepsis alert systems in emergency departments found lower mortality overall (RR 0.81) and particularly for electronic alerts (RR 0.78), but also emphasized heterogeneity, non-randomized evidence, and the persistent problem of false positives [35].

Across current applications, the most credible near-term role of AI in infectious-disease care is workflow acceleration, not autonomous clinical replacement. The evidence is strongest where AI operates at a clear bottleneck in care delivery, such as rapid radiographic triage, early discrimination of host-response patterns, or point-of-prescribing stewardship support [38]. In these settings, the practical value of AI lies in shortening time to confirmatory testing, prioritizing limited diagnostic capacity, or reducing mismatched empiric therapy. Tuberculosis chest-radiograph triage remains the most mature example of this pattern [39,40]. The reviewed literature consistently positions TB computer-aided detection as a prioritization tool in high-volume settings rather than as a stand-alone diagnostic endpoint. By contrast, sepsis prediction illustrates a more cautionary lesson: good internal or vendor-reported performance does not guarantee transportability, and external validation may reveal lower discrimination, reduced sensitivity, and a clinically important alert burden [27]. This contrast is important because it shows that “AI in infectious diseases” is not a single evidentiary category; readiness depends heavily on task definition, workflow context, and the stability of the data-generating environment [23].

Diagnostic and treatment-facing applications between these two poles include host-response testing, MALDI-TOF-based antimicrobial-resistance prediction, and stewardship-oriented ordering tools [34]. These approaches are mechanistically attractive because they may shorten the interval between specimen acquisition and action or improve empiric prescribing. However, their evidentiary status is more heterogeneous than that of TB triage. The literature suggests promise, but the main question is no longer whether such tools can generate informative predictions in selected studies; it is whether they deliver reliable, generalizable, and actionable benefit under routine clinical conditions.

Treatment-support systems appear most useful when they are embedded in clinician-facing stewardship workflows rather than positioned as independent recommendation engines. The most persuasive examples are those that combine individualized resistance forecasts with guideline-aware ordering logic, because their outputs are linked to clinically meaningful decisions: reduced mismatch between empiric therapy and likely susceptibility, less unnecessary broad-spectrum exposure, and potentially earlier adaptation of therapy [36]. Even here, however, benefit remains context-dependent and is strongly shaped by clinician uptake, local guideline alignment, and workflow integration.

AI in infectious disease diagnostics is most useful when presented as a set of clinically embedded support tools rather than as a monolithic technology. Current applications span image-based triage, structured EHR prediction, microbiology and laboratory augmentation, and host-response diagnostics. However, the literature is heterogeneous in both methodological rigor and deployment maturity. Some systems remain retrospective proof-of-concept models, whereas others have undergone external validation, prospective multicenter evaluation, or regulatory review. Organizing the evidence by clinical task and implementation maturity clarifies where AI is currently actionable and where findings remain preliminary [37].

## 5. Clinical Workflows, Integration, and Performance Evaluation

Raw model discrimination is less crucial to clinical impact, and predictions can be used in care at a variety of locations and in a variety of ways. In infectious diseases, the application of AI integration can be viewed as a closed-loop socio-technical system in data capture, inference, human interpretation, action (antibiotics, isolation, and workup), outcomes, and feedback/model monitoring [41]. An example with a good documentation is sepsis early warning. The implementation of the Sepsis Watch describes an actual system application of deep learning into standard practice, focusing on multidisciplinary governance, software architecture, and workflow optimization instead of a model-only system [42]. Additional qualitative studies point to obstacles to use, e.g., trust, alert fatigue, and the necessity to establish clear responsibility boundaries, and demonstrate that effective use requires model outputs to be balanced with the current clinical practices and escalation procedures [43]. The performance and standard care should be evaluated in three directions, such as diagnostic accuracy (AUC, sensitivity/specificity, calibration), clinical timeliness (time-to-diagnosis, time to appropriate therapy), and patient and system outcomes (mortality, length of stay, antibiotic exposure, resistance pressure, resource utilization) [44,45].

One of the warning indicators is that proprietary tools commonly used are externally validated. A single large external validation of deployed sepsis prediction model had significantly worse discrimination than is reported in vendor documents (hospitalization-level AUC 0.63) and low sensitivity (33%) and has calibration and possible clinical implication concerns [8]. It applies directly to infectious diseases since epidemiology, testing behavior, and treatment patterns change regularly, so the threat of dataset drift is of first order. Beyond headline discrimination metrics, the clinical behavior of infectious-disease AI systems depends strongly on how models are built. Sepsis models commonly combine static covariates with dynamic time-updated vital signs and laboratory variables, but they differ substantially in temporal representation, thresholding, and handling of out-of-distribution data. For example, recurrent models such as Sepsis Watch rely on continuously updated EHR inputs, whereas COMPOSER explicitly weights variables by the time since last measurement and uses conformal prediction to reject unfamiliar or low-quality inputs rather than forcing a prediction [42]. Commercial sepsis tools may also differ in model family; for instance, newer implementations of the Epic Sepsis Model use gradient-boosted trees and allow local fine-tuning [8]. In antimicrobial-resistance prediction, performance depends not only on the classifier itself but also on spectral preprocessing, calibration, class balance, and feature interpretation. MALDI-TOF studies highlight how binning, normalization, calibrated classification, and feature-importance analysis can materially influence transportability. Thus, reported AUCs should be interpreted in the context of predictor selection, missingness handling, threshold calibration, and explainability rather than as stand-alone markers of clinical readiness.

**Figure 3:**
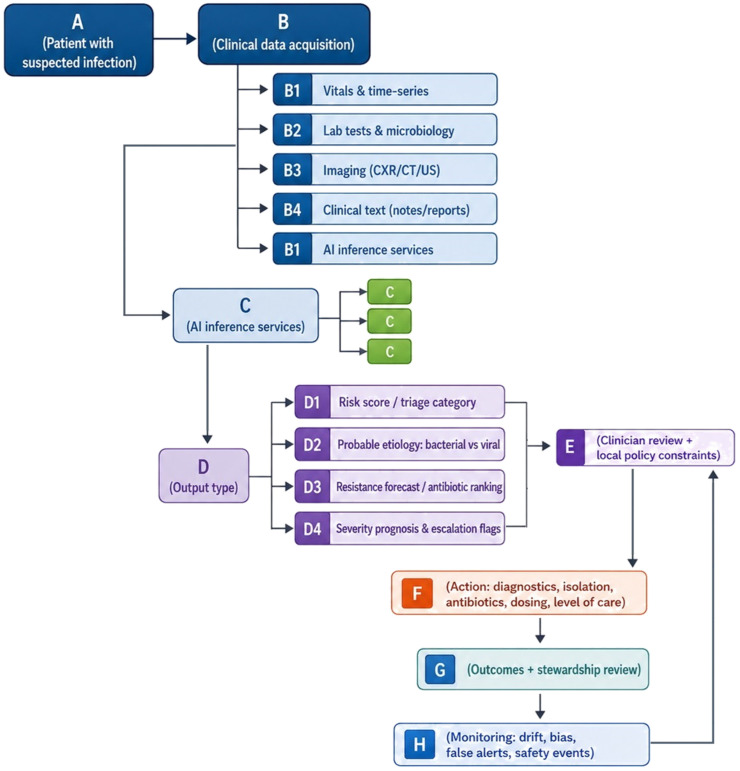
Conditions for safe clinical deployment of AI in infectious diseases: data capture, model inference, clinician interpretation, action, outcome monitoring, and drift governance. Clinical and laboratory data feed into AI algorithms (ML, NLP/LLM) that output recommendations (C). These may trigger actions (D) like clinician review (E) and ultimately improving patient outcomes (F) and monitoring (G).

Operational requirements are interoperability and auditability. As an example, the public-health reporting infrastructures adopt modern data-exchange standards (e.g., Fast Healthcare Interoperability Resources—FHIR) to have a direction of automating surveillance, and reducing reporting load, a direction also conducive to AI, by enhancing data completeness and standardization [46,47] (Figure 3).

Some types of infectious-disease AI tools are sold as regulated medical devices, and many others (in particular, local EHR-based decision support) are not sold as regulated medical devices or are sold under policy categories in which regulatory obligations vary by jurisdiction and desired application (Table S2). In the United States, regulators publish an “AI-enabled medical devices” list and device-specific decision summaries (e.g., 510(k), De Novo) that describe intended use and evidence expectations [48,49,50,51,52,53]. The recent lifecycle guidance of the same regulator highlights transparency, risk management in the context of a total product lifecycle, and optional, though encouraged, structured disclosures, including model cards, to convey intended use, data characteristics, and constraints [54].

In a 2021 head-to-head study in Bangladesh with 23,954 CXRs of presumptive TB patients, five commercial AI algorithms were trained to interpret the images and were compared with radiologists. All of them were quite accurate (AUC of 0.85–0.91) and far better than human readers [22]. Practically, these tools might reduce by half the amount of costly Xpert tests yet still have a sensitivity > 90%, thus triaging patients faster with molecular confirmation. The majority of these Computer-Aided Design (CAD) products are commercially available (e.g., Conformite Europeenne, CE marked in some countries), and the prime clinical advantage is the priority-based testing as opposed to final diagnosis. A retrospective study of 38,455 hospital stays in the United States of a commonly used proprietary sepsis model (Epic Sepsis Model) demonstrated poor performance [8]. The overall AUC of the model was 0.63 (hospital-level), and the sensitivity and Positive Predictive Value (PPV) were approximately 33 percent and 12 percent, respectively, which implies that the model missed the majority of sepsis cases and produced numerous false alarms [8]. This underscores the danger of implementing not adequately validated models and alert fatigue in practice. In comparison, a quality-improvement report (the Sepsis Watch project) in 2020 was devoted to the procedure of including the deep learning sepsis warning system into care [55,56]. The same study did not highlight the AUC but reported the relevance of workflow redesign, involvement of clinicians, and the use of last-mile alert routing to realize the earlier detection of sepsis. Etiologic guidance is earlier with the promise of new molecular and ML tests. A prospective cohort of 688 emergency departments (ED) patients in 2025 employed a 29-mRNA host-response test (Inflammatix BVN/SEV) to differentiate between bacterial and viral infections and 30-day mortality. It had AUC 0.76 bacterial vs. non-bacterial, 0.89 viral vs. non-viral and 0.77 30-day mortality [57]. Theoretically, such transcriptomic tests can lead to therapy prior to culture results (e.g., by indicating early escalation or de-escalation). Likewise, MALDI-TOF mass spectra with ML have been demonstrated (2022) to anticipate antibiotic resistance far quicker than culture [28]. Weis et al. in *Nature Medicine* show AUCs for important pathogens (e.g., 0.80 of Staphylococcus aureus) are about 0.74–0.80, with culture-based AST requiring approximately 72 h [27]. Such ML methods (still largely in the research/validation phase) would potentially provide resistance flags a day or longer before existing methods. A large deployment of an AI-augmented UTI order set (“UTI Smart Set”) also showed impressive results [35]. Physicians adhering to the AI resistance predictions and guideline-based recommendations in a national outpatient network had significantly lower antibiotic mismatch rates (8.9% vs. 14.2% of cases), and the use of fluoroquinolone (ciprofloxacin use 6.4% vs. 32.9% of cases) was drastically reduced [35]. This ML-based clinical decision support successfully steered clinicians to narrow-spectrum decisions upon prescription to help minimize inappropriate exposure and help with stewardship (Table 1).

In these instances, AI systems tend to outperform or speed up typical care in triage or decision support, although a potential difference in practice is context-dependent. Image triage (TB X-rays) is always faster than human readers. Without close attention to validation, predictive models (sepsis alerts) can fail. It is important to integrate AI into workflows (as in the case of “Sepsis Watch” or UTI order sets) to obtain a quicker response and clinician buy-in. Most tools are still in pilot or research phase, and there are very few fully FDA-cleared products; the more mature TB-CAD and some mRNA tests. The reported gains in each instance are mostly earlier or more specific interventions (swift triage to conclusive tests, swifter antibiotic changes) as opposed to fresh diagnoses as such. All these studies (Table S3) emphasize that AI has the potential to substantially increase performance measures and possibly patient outcomes (e.g., reduced mortality in the AI-CDSS trial) [34,58,59]. However, effective implementation needs the strong assessment, incorporation, and in many cases regulatory controls before general practice (Table 2).

A recurring theme across infectious disease AI is that strong internal discrimination does not guarantee safe generalization across hospitals, patient groups, or clinical workflows. This is evident in tuberculosis screening, where product performance and optimal thresholds vary across age, HIV status, and prior TB history, and in sepsis prediction, where an independently validated proprietary model showed materially weaker performance than internal or vendor-reported estimates [25]. Accordingly, studies that include temporal validation, independent external cohorts, subgroup analysis, calibration assessment, and workflow-aware threshold selection should be weighted more heavily than single-center retrospective model-development reports.

## 6. Data Requirements, Datasets, and Validation Standards

Infectious-disease AI has an abnormally large data appetite since the presence of pathogen, host response, disease severity, and treatment response can be out of phase. This generates reiterating information needs: powerful reference criteria (microbiology, adjudication groups, or clinically significant composites), in particular where labels are incomplete (e.g., suspected infection). The need to conduct a study on host-response is usually necessitated by the fact that infection attribution is unclear [29]. Infectious syndromes are changing at a rapid pace; models require time-stamped characteristics and a keen horizon delineation (e.g., risk in 24 h). Tests, resistance practices, and admission patterns differ among hospitals and regions, external validation is thus not incidental, and it is at the core of safety [8]. High-volume critical-care EHR data is extensively applied to infectious-risk prediction (such as sepsis risk and multidrug-resistant organisms—MDRO risk) since it comprises high-density vitals/labs, diagnoses, and treatments. MIM-IC IV offers de-identified Intensive Care Units (ICU) and ED data on a large-scale, and is generally utilized to build and test clinical prediction models, yet it needs credentialing, training, and a data use agreement [58]. On the same note, the eICU Collaborative Research Database offers high granularity and large sample size multicenter ICU data, which is useful in external validation and heterogeneity studies [60]. The NIAID TB Portals program disseminates infectious-disease-specific multimodal TB research, such as imaging (chest X-ray/computed tomography), genomics, drug susceptibility testing, and rich clinical metadata, including manual and model-generated annotations, to support diagnostic as well as AMR-related research [61]. DRIAMS is a publicly accessible large MALDI TOF mass spectra dataset, which is associated with antimicrobial resistance phenotypes in several different institutions, and it can be used to conduct AMR prediction and transferability analyses [62]. One of the issues that have continued to hinder reliable translation is the lack of or partial reporting of AI interventions in clinical trials. There are now consensus reporting extensions to major study types: CONSORT-AI when conducting a clinical trial with AI interventions, as its primary concern is to provide a clear description of the AI system, its integration, and the management of errors and human–AI interaction [63]; SPIRIT-AI for AI clinical trial protocols (what must be specified before running the study) [64]; and TRIPOD + AI to predict model development/validation report with regression and ML, to deal with the increased complexity of today’s predictive systems [65].

**Figure 4:**
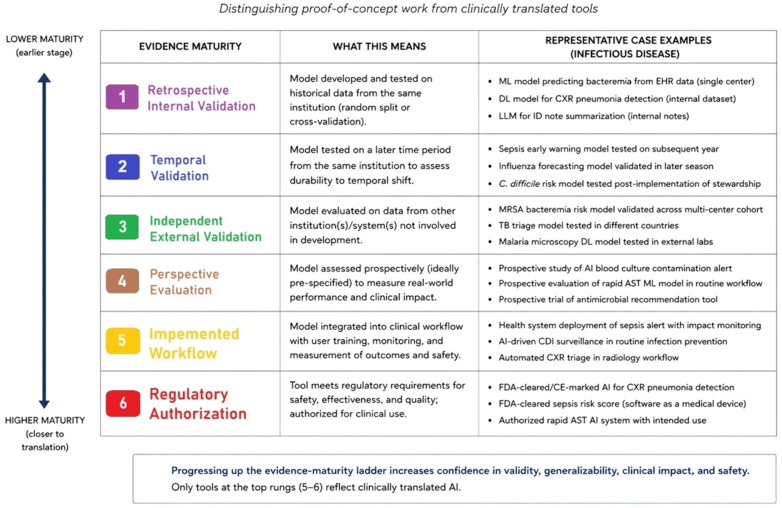
Evidence-maturity ladder from development to translation—progression from retrospective internal validation to regulatory authorization, with lower maturity at the top, higher maturity at the bottom, and columns explaining each level and giving infectious-disease case examples. The main takeaway is that confidence in validity, generalizability, clinical impact, and safety increases as a tool moves down the ladder, with only the highest rungs representing clinically translated AI.

A recurring theme across the literature is that model discrimination alone is not sufficient for clinical impact. In infectious diseases, AI functions within a socio-technical system that includes data capture, inference, clinician interpretation, escalation pathways, treatment action, outcome measurement, and ongoing monitoring. The reviewed evidence therefore supports evaluating AI in at least three dimensions: technical performance, time to action, and patient- or system-level outcomes. External validation is especially important in infectious disease contexts because pathogens, testing platforms, hospital workflows, and antimicrobial-resistance ecologies change over time and across sites (Figure 4). Sepsis prediction is the clearest example in the current literature of how performance may deteriorate during external evaluation, with consequences for actionability and alert fatigue. This lesson should be interpreted broadly: models embedded in dynamic clinical ecologies require local calibration, threshold review, and post-deployment monitoring if they are to remain safe and useful [66].

Dataset shift should be treated as an expected operational reality rather than an occasional technical nuisance. A recent systematic review of dataset shift in health prediction models found that temporal shift and concept drift are among the most frequently encountered problems; while monitoring strategies, statistical drift detection, retraining, and feature engineering are the most common mitigation approaches. Importantly, the same review noted limited external validation and limited real-world integration of these solutions, suggesting that future infectious disease AI deployments should pre-specify monitoring dashboards, recalibration triggers, and retraining governance before implementation rather than after performance deteriorates [67]. Recent consensus recommendations from the STANDING Together initiative argue that health datasets should be transparently documented for representation, missingness, and population limitations, and that AI systems should be evaluated proactively across demographic groups [68]. This is directly relevant to infectious diseases, where subgroup variability has already been observed in TB CAD studies, while some newer host-response diagnostics have reported more stable performance across racial groups than conventional biomarkers. The practical implication is that infectious disease AI reviews should report not only pooled AUROC values but also subgroup results wherever available, especially across race, age, sex, geography, immunocompromised status, HIV, and previous disease history [69].

## 7. Cost-Effectiveness of AI in Infectious Diseases

The health-economic literature on AI in infectious diseases is encouraging but still methodologically immature. Many studies suggest that AI-enabled tools may be cost saving or cost-effective by reducing downstream testing, shortening length of stay, limiting unnecessary antimicrobial exposure, or improving treatment targeting. However, most of the available evidence is derived from decision models or simulations, often under simplifying assumptions that may not fully capture drift, implementation costs, training needs, or heterogeneity across settings. For that reason, current economic evidence should be interpreted as supportive but provisional. The practical message is not that AI has already been shown to be universally cost-effective, but that certain applications warrant local evaluation because they have plausible value mechanisms. Procurement and adoption decisions should therefore emphasize total cost of ownership, implementation requirements, and prospective monitoring rather than relying only on model-based incremental cost-effectiveness ratios.

Emerging evidence suggests that AI-enabled tools in infectious disease care often improve outcomes at modest incremental cost. Many modeling studies find AI interventions are cost-saving or yield very low incremental cost-effectiveness ratio (ICERs) [69,70]. For example, AI triage for tuberculosis (TB) in Pakistan dominated standard care (cost per disability-adjusted life year, DALY ~$39) [71], and AI-monitored TB therapy in the US halved per-patient costs while slightly improving Quality-Adjusted Life Years (QALYs) [66]. However, most analyses are context-specific and rely on simplifying assumptions (e.g., static models, omitted indirect costs), so the reported benefits may be overstated [22,43]. Policy-makers should demand rigorous, locally adapted health-economic evaluations and post-market monitoring to confirm real-world value.

AI-based chest X-ray triage (with reinforced follow-up) dominated smear/Xpert testing. It reduced lab tests by ~74% and saved ~$4383–$12,637 per 1000 patients, averting ~13–15 DALYs per 1000. This implies a cost per DALY averted of only ~$39–$40 (far below Pakistan’s ~US$195/DALY threshold) [59]. “AiCure” video-observed therapy was cost-saving versus in-person Directly Observed Therapy (DOT) [51]. Over 16 months, AiCure cost $2668 and yielded 1.05 QALYs per patient versus $4894 and 1.03 QALYs for standard DOT (ICER dominant). The net saving (~$2226) came mainly from avoiding nurse travel and coordination [72]. An ICU ML algorithm forecasting sepsis onset dominated usual care. In a model of Swedish ICUs, the algorithm reduced costs by ~€76 per patient (primarily via 0.16 fewer ICU days) and improved survival (356 deaths avoided/year) [73]. It was “dominant” (cost-saving) in most scenarios and far below the €20,000/QALY threshold. A United States (US) study modeled an ML tool to find undiagnosed hepatitis C in outpatient Electronic Medical Records (EMRs). At optimal sensitivity (40%), the tool cost ~$96.90 more per patient but gained 0.0011 QALY, yielding an ICER ~$92,245/QALY (below a $100,000/QALY WTP) [74]. This was judged cost-effective, illustrating that even at large scale AI can meet standard thresholds. Finally, a 2025 Indian analysis (Raval et al.) evaluated two AI-CXR tools for TB screening. One tool (qXR) was cost-saving (ICER ≈ −$120 per case) and the other (Genki) had an ICER of about $137 per screened case, both well below India’s per capita GDP. A recent review of 19 economic evaluations (across specialties) found that clinical AI often yields ICERs well under accepted thresholds [75]. Most reported both improved QALYs and lower costs (by reducing unnecessary procedures). For example, AI screening in other domains saw ICERs of only a few thousand USD/QALY [75]. These promising results (often dominant interventions) suggest AI can be highly cost-effective, though infectious-disease data remain limited (Table 3).

There are three motivations behind AI in the context of infectious care [44]. Better sensitivity/specificity result in more true positives and fewer false positives, and less waste on tests and treatments [75]. As an example, increased sensitivity of TB triage or DR screening significantly reduced downstream expenses [73,74]. Quick diagnosis and specific treatment minimize hospital stay and visits [78,79]. The sepsis algorithm saved days in the ICUs by allowing 3 h earlier treatment [80,81]. AI also reduced the number of confirmatory tests (TB CXR triage reduced microbiology by approximately 74%), and nurse hours (video-DOT eliminated home visits). The development of AI and per-use costs can be offset with savings [80]. Economic models focus on how AI needs to be cheap at scale (e.g., less than US$19 per colonoscopy procedure) to be cost-saving [81]. Other drivers are training, integration and maintenance expenses. The fixed costs can be spread out over time providing a better cost-effectiveness with increased deployment (greater patient volumes).

Decision models or simulations (mostly, not time-varying) are used in most studies, and they can overestimate benefits (failing to reflect learning curves, or decreasing performance) [82]. Data infrastructure, training of providers, and regulatory compliance are important costs that are not usually considered. Also, it is possible to have algorithmic bias: the training data can fail to represent all populations, and AI could be effective in a particular environment and ineffective in another. There is a great variety of heterogeneity: both high-income (United States of America, Sweden) and low-income (Pakistan) settings have been used as examples, and thus, there is no guarantee that results will be similar across countries or diseases [11,72,76]. Overall, the reported gains may be context-dependent. There are dynamic factors (data drift, new variants, software updates) and ethical/regulatory costs (validation, privacy) which introduce additional uncertainty not often quantified by current studies. The decision-makers ought to incorporate economic and equity-based criteria in procurement and governance to realize the potential of AI [83,84]. The buying process must demand both clinical and economic documentation (e.g., Health Technology Assessment—HTA or pilot data) and take into account the total cost of ownership (including licenses, hardware and training) [85,86,87]. A standardized model of assessment of AI tools should be implemented in health systems (e.g., Consolidated Health Economic Evaluation Reporting Standards for Interventions that use AI, CHEERS-AI reporting). Notably, implementers should observe real-world impact: measure diagnostic accuracy, outcomes, and resource use following deployment and change policies as necessary [88]. Protective measures on equity are necessary. The World Health Organization (WHO) principles of AI focus on its transparency, strict validation, and mitigation of bias in health [89]. To illustrate, regulators or payers might require AI vendors to disclose dataset demographics and performance by subgroup, and to perform equity audits at the end of implementation. Teamwork among clinicians, engineers and ethicists can also guarantee the adoption of AI has training programs and supervision [90]. Lastly, incentives can be aligned and risks controlled with adaptive financing (e.g., outcome-based contracts or staged rollouts with interim CE analysis) [91]. The innovations of AI-driven diagnostics and care can represent a high value in infectious disease management, and in many cases, present the same or even better patient outcomes at a reduced cost. The important success factors are good diagnostic accuracy, quick turnaround, and close adherence in the care pathways. However, existing literature is not comprehensive and can exaggerate benefits because of the modeling assumptions. Going forward, stake-holders should insist on quality and context-specific evaluations and sustained post-market evaluation. The theoretical cost-efficiency of AI can only be translated into actual health benefits through strict scrutiny, open leadership, and implementation of the theory which is concerned with equity.

## 8. Generative AI and Large Language Models in Infectious-Disease Care

Beyond the narrow-AI applications discussed above, the past three years have seen rapid uptake of generative AI in infectious-disease (ID) practice. Large language models (LLMs) such as GPT-4, GPT-4o, Gemini, and Claude differ from earlier task-specific classifiers in three respects relevant to ID: they accept free-text clinical input and produce free-text output without bespoke feature engineering; they can be conditioned at inference time through prompting and external knowledge integration rather than retraining; and the most recent releases are multimodal, jointly reasoning over text, imaging, and structured data within a single model. The pace of adoption is striking—a recent ID-focused review noted that a PubMed search for the term “ChatGPT” returned several thousand records for 2024–2025 alone [92], and a systematic review identified rapidly growing application of natural language processing and LLM tools across pneumonia detection, invasive mold diagnosis, bloodstream infection management, and HIV care, while flagging the early-stage and fragmented nature of the evidence [93]. For ID, where a large amount of unstructured information (admission notes, microbiology comments, antimicrobial stewardship audit records, guideline documents) must be synthesized at the point of care, this shift is potentially transformative—but it also creates failure modes that the narrow-AI safety literature does not fully address.

Evidence for generative AI in infectious-disease practice remains early phase and should be interpreted more cautiously than the fluency of current models might suggest. Much of the available literature consists of vignette studies, retrospective case comparisons, bounded classification tasks, and small single-center pilots. These studies are useful for feasibility testing and hypothesis generation, but they do not yet establish that general-purpose large language models improve time to appropriate therapy, antibiotic exposure, mortality, or other patient-centered outcomes in routine infectious-disease care. The practical implication is that generative AI should presently be framed as a supportive, bounded layer rather than as an autonomous infectious-disease consultant. Its most defensible near-term roles include summarizing clinical context, surfacing guideline-concordant options through retrieval-augmented workflows, organizing documentation, and contextualizing outputs from narrower diagnostic or stewardship models for human review. The evidence base is not yet sufficient to justify unmonitored or unsupervised treatment-facing deployment, particularly in settings where hallucination, missing context, or overly confident antimicrobial advice could cause harm.

Several studies have evaluated LLMs as ID consultative aids. An early *Lancet Infectious Diseases* evaluation of ChatGPT on eight infection scenarios found that the model produced fluent, scenario-aware summaries but gave inconsistent and sometimes unsafe antimicrobial recommendations, and could not request the additional information a human consultant would normally seek [94]. A 2025 comparative study of GPT-4o against three resident physicians and three ID specialists across 75 questions reported that GPT-4o achieved comparable accuracy to specialists on true/false questions (87.5% vs. 90.3%) but specialists significantly outperformed GPT-4o on open-ended (*p* = 0.008) and clinical-case questions (*p* = 0.02); GPT-4o was, however, significantly more complete than residents on open-ended and case-based questions [95]. A retrospective pilot at Saint Vincent Hospital comparing physicians with ChatGPT-4 on 50 cases of septic shock and severe pneumonia similarly found that physicians selected more appropriate investigations and antibiotics, although concordance was higher for pathogen-specific coverage of multidrug-resistant organisms [96]. When LLMs are scaffolded with chain-of-thought prompting and external knowledge integration, performance improves substantially: a 2024–2025 prospective study at a 399-bed Veteran Affairs Medical Center deployed a GPT-4 chatbot with locally uploaded BCID2-interpretation guidelines and reported recommendations broadly comparable to formal antimicrobial stewardship audit and feedback in 43 consecutive cases of bacteremia [97]. For the narrower task of antimicrobial classification across 7239 medications from the CHARM project, four LLMs were tested with one round of feedback-driven correction; Gemini 2.5 Flash and Claude Sonnet 4 reached 99.6% and 99.4% accuracy, respectively, while ChatGPT-3.5 and Copilot remained at 81.0% and 79.7%, suggesting that well-bounded LLM tasks within stewardship workflows are now within reach for the strongest current models but that model selection matters [96]. A retrospective LLM-augmented chart review of seven sepsis cases in an Italian ID unit similarly showed plausible guideline-aligned recommendations on antibiotic therapy, isolation, and device management, while flagging incomplete documentation as the principal practical limitation [98].

The dominant safety concern specific to generative AI is hallucination—fluent, confident output that is factually wrong, fabricated, or unsupported by the underlying evidence. In ID, where small errors in pathogen, drug, or dose can be dangerous, this is not theoretical: editorial commentary in *Clinical Infectious Diseases* has described LLMs as a “black box” that should not yet be trusted for autonomous antimicrobial advice, citing frequent confabulation, lack of contextual awareness, inscrutable training data, and propensity to recapitulate biases [99], and Australian ID groups have called explicitly for evidence generation and regulatory frameworks before clinical deployment [100]. The leading mitigation strategy is retrieval-augmented generation (RAG), in which the LLM is constrained to answer only on the basis of documents retrieved from a curated knowledge base such as national stewardship guidelines, local antibiograms, or IDSA documents [101]. Recent work in ID-relevant public-health question answering has shown that multi-evidence RAG architectures combining dense retrieval, BM25 keyword retrieval, and biomedical knowledge graphs can reduce hallucination rates by more than 40% relative to stand-alone LLMs, while reaching accuracy and F1 scores of approximately 0.79 [102]. A 2025 PRISMA-style systematic review of 30 RAG in healthcare studies, however, identifies retrieval noise, domain shift, generation latency, and limited explainability as persistent barriers, and notes that very few RAG-enabled clinical systems have undergone prospective validation [103].

A second strand of generative AI directly relevant to ID is the emergence of multimodal foundation models that jointly process medical images and text. Vision–language foundation models for chest radiography and 3D computed tomography have demonstrated zero- or few-shot detection of pneumonia, tuberculosis, and COVID-19-related abnormalities, with reported performance approaching task-specific models trained on much larger labeled cohorts; these systems also enable interactive radiology report drafting and could be integrated upstream of the TB-triage workflows discussed in earlier sections [82]. In parallel, biological-sequence LLMs—protein, DNA, and RNA language models—have been adapted to ID-relevant tasks including pathogen identification, evolutionary surveillance of viral variants, host–pathogen interaction prediction, and antimicrobial-peptide and vaccine-component design [103]. These models extend the scope of “AI in ID” beyond bedside decision support into pathogen surveillance and therapeutic discovery, but introduce additional governance questions about biosafety and dual-use risk that conventional medical-device frameworks do not currently cover [103]. Three observations follow from the literature surveyed above and align with the broader themes of this review. First, the evidence base for LLMs in ID is dominated by retrospective vignette studies, single-center pilots, and commentary; randomized or pragmatic trials with patient-centered endpoints (time to appropriate therapy, antibiotic exposure, mortality) are essentially absent—a gap that mirrors but is more acute than the one identified earlier for narrow AI [4,100]. Second, the regulatory landscape is still consolidating: the WHO 2024 guidance on the ethics and governance of large multimodal models for health frames LLMs as high-stakes systems requiring transparency, dataset disclosure, and post-deployment monitoring [6]; the FDA lifecycle guidance on AI-enabled device software functions extends predicate-device thinking to model updates and drift [37]; and the EU AI Act classifies most clinical LLMs as high-risk systems with attendant conformity obligations under MDR/IVDR [104,105]. Third—and most relevant for the integration story of this review—LLMs are unlikely to replace the narrow-AI tools described in earlier sections (TB CXR triage, sepsis prediction, MALDI-TOF resistance forecasting, host-response classifiers). Rather, the realistic near-term role is as an interpretive layer that summarizes and contextualizes the outputs of those underlying models for clinicians, integrates them with local guidelines through RAG, and produces structured, auditable recommendations that fit into existing stewardship and escalation workflows [97,100,101]. Demonstrating that this combined workflow improves time to action, antibiotic appropriateness, and patient outcomes—rather than merely generating plausible text—is the principal evidence-generation task for the next several years.

## 9. Limitations, Risks, Ethics, and Implementation Barriers

The generalizability and drift are particularly acute in the case of infectious diseases due to the fact that the prevalence of pathogens, resistance patterns, and testing strategies vary over time. The failure in external validation of sepsis prediction exemplifies that excellent internal performance does not imply that it can be relied on in practice at different sites and different times [8]. Whenever training data fails to represent certain groups, devices or different care settings, bias and representativeness ensues. This risk is specifically identified in the lifecycle-based regulatory guidance, which states the systematic error risks to be created by biased datasets and overfitting to site/scanner artifacts [55]. Security and adversarial threats are not a hypothetical concept: on medical imaging DL systems, adversarial perturbations can be exploited to open a backdoor to adversarial manipulation of outputs and inputs, leading to false outputs. A radiological systematic review provides a summary of types of attacks and clinical implications, which highlights the importance of cybersecurity when AI Infectious-disease data is commonly high-stakes: the data incorporate medical information with potentially stigmatizing conditions (e.g., TB, human immunodeficiency virus—HIV) and may have implications for public health [106]. According to the General Data Protection Regulation (GDPR), the data related to health are special-category personal data, and the restrictions on their processing are stringent and the exceptions are limited; legal grounds and protection should be pronounced [106]. The Health Insurance Portability and Accountability Act (HIPAA) Privacy Rule of the United States comprises national standards of confidentiality of individually identifiable health information (PHI) possessed by covered entities and business partners [107]. Based on an AI-specific regulatory approach, the EU AI Act framework on high-risk systems incorporates risk mitigation, high-quality data to decrease discriminatory results, logging and documentation for traceability, human oversight and robustness/cybersecurity [105,106]. An instruction on the interaction with EU medical device regimes (medical devices and in vitro diagnostic medical devices regulations, MDR/IVDR) makes it clear that AI Act high-risk classification corresponds to the underlying device classification and brings parallel conformity and monitoring issues into play instead of automatically modifying MDR/IVDR risk classification [108].

The implementation is the point at which several models that seem to be clinically impressive fail. High false-positive rate or bad ways of presentation may result in clinicians not paying attention to alerts; sepsis deployments underline the importance of routing and escalation reasoning as well as continuous fine-tuning [43]. Thorough unstructured data flows (labs, vitals, meds) and imaging interconnections are missing, model inputs are subpar and outputs are unreliable [47,48]. Due to licensing, integration, monitoring, and retraining, recurring costs arise; health systems should have total cost of ownership models and transparent measures of the benefit. AI outputs may provide a new degree of ambiguity in terms of responsibility; they should explicitly define to whom the responsibility to act (or not act) on alerts and recommendations lies [52,109].

The central finding of this review is that AI in infectious diseases is not limited by model generation alone; it is limited by external validity, implementation quality, and outcome-based evidence. The comparative maturity of different applications matters. Tools tied to relatively constrained modalities and well-defined workflow bottlenecks, such as TB radiograph triage, appear closer to routine augmentation. Tools operating in dynamic syndromes and rapidly shifting hospital ecologies, such as sepsis prediction, require more conservative deployment and more aggressive monitoring. Generative AI introduces a related but distinct challenge: greater flexibility and synthesis capacity without yet adequate prospective evidence of safe and clinically meaningful impact.

Three priorities emerge for translation into routine care. First, externally validated and locally calibrated performance must be demonstrated in populations and workflows similar to those in which the tool will be used. Second, prospective or pragmatic studies should evaluate clinically relevant endpoints such as time to action, antibiotic appropriateness, downstream testing, and patient outcomes, rather than relying mainly on AUC or related technical metrics. Third, governance frameworks must specify accountability, drift monitoring, human oversight, and documentation standards. Without these elements, AI may increase workload and uncertainty rather than improve care.

This review has several limitations. It was designed as a structured narrative synthesis rather than an exhaustive systematic review, and therefore does not claim full capture of all eligible studies. The evidence base also varies substantially by domain, with comparatively mature literature in narrow image-based triage and less mature evidence in generative AI. Nonetheless, the deliberate emphasis on external validation, implementation relevance, and official regulatory or guidance documents improves the practical utility of the synthesis and responds directly to the gap between technical performance claims and real-world clinical readiness.

## 10. Future Research Directions and Recommendations for Clinicians and Researchers

Priorities that are particularly useful during infectious disease situations are: the pragmatic trials with high priority that quantitatively measure time to appropriate therapy, antibiotic exposure, and mortality; the uncertainty-sensitive systems that can withhold information and therefore can abstain (“I don’t know”); the robust external validation across regions and resistance ecologies; the privacy-aware learning (federated learning) to enhance generalizability without centralizing sensitive data—an approach already reviewed specifically for infectious-disease contexts, though most published work still concentrates on COVID-19-era data rather than broader pathogens [82,93,106,110].

There are several recommendations in the case of clinicians (implementation and safe use) [111,112,113,114,115,116,117,118,119]:

- The use of AI outputs as support for decision-making, not diagnosis. Mandate that it is clearly documented how they are to be used, the known failure modes, as well as the local policy for escalation of action.
- The selection of externally validated AI systems in environments with similar patients (patient mix), lab workflow, and imaging equipment and published calibration/threshold selection logic.
- The selection and tracking of operational indicators on a regular basis: alerts, clinician response rates, false positives, and time to antibiotics, and downstream testing load, in particular for sepsis and syndromic alerts.
- The development of a human factors safety layer: understandable UI, clarification where possible, and an escalation channel which obviates alert fatigue (e.g., tiered or routed alerts).
- The establishment of incident response policies (including cybersecurity): clarifying what occurs in the event of the suspected malfunction, drift, or adversarial interference of an AI tool.

In case of researchers (quality of evidence and translational science) [114,115,116,120]:

- The study designs should be based on clinical decisions and clinical outcomes (appropriate antibiotics, de-escalation, length of stay, mortality) and not only AUC; time to action endpoints should be used where the model purports to have the advantage of speed.
- The reporting and use standards (CONSORT-AI, SPIRIT-AI, TRIPOD + AI), with a clear position on missingness, drift, and human–AI interaction.
- The development of a generalizability plan: a registry of external validations at hospitals/regions, and stratification of performance based on clinically meaningful subgroups (age, comorbidity, immune status, and device/vendor).
- The measurement of uncertainty and predictability; to apply abstinence levels of out-of-distribution cases (novel pathogens, new assays, changed admission behaviors).
- The compliance with the privacy and governance first-class design considerations: seek federated or privacy-conscious learning where feasible and ensure lawful processing of health data under appropriate legal frameworks.

## 11. Conclusions

Current evidence suggests that AI is already clinically meaningful in infectious diseases when it functions as an augmentation layer within well-defined workflows, particularly for high-volume triage, host-response supported diagnosis, bloodstream infection prediction, and antimicrobial stewardship. Nonetheless, the field remains uneven: many models are still retrospective and single-center, whereas only a smaller subset has progressed to external validation, multicenter prospective study, real-world implementation, or regulatory authorization. Future research should therefore prioritize multicenter prospective evaluations, standardized reporting of discrimination, calibration, PPV and NPV, subgroup fairness auditing, explicit drift-monitoring and recalibration plans, interoperable integration with EHR and laboratory systems, and regulatory-concordant post-market surveillance. This pathway is more likely to produce safe and generalizable clinical translation than continued proliferation of isolated proof-of-concept models. Framed this way, AI in infectious diseases is a tool for disciplined augmentation, not clinician replacement.

## Figures and Tables

**Table 1:** Representative studies and performance.

Domain	Representative Example	Most Decision-Relevant Signal	Evidence Maturity	Main Caution	References
TB CXR triage	Commercial CAD tools for presumptive TB screening	AUC approximately 0.85–0.91; rapid prioritization to confirmatory testing	Comparatively mature	Best viewed as triage, not final diagnosis	[22]
Sepsis prediction	EHR-based early-warning models	External validation may fall to AUC 0.63, sensitivity 33%, PPV 12%	Mixed and fragile	Poor transportability and alert burden	[8]
Host-response and AMR prediction	29-mRNA classifiers; MALDI-TOF-based resistance prediction	Earlier etiologic or resistance-informed action; AUCs in the 0.74–0.89 range depending on task	Promising but heterogeneous	Assay-, site-, and population-dependence	[57]
Stewardship decision support	AI-guided UTI order sets; AI-CDSS	Lower mismatch with recommendations; less unnecessary broad-spectrum exposure	Promising real-world workflow evidence	Benefit depends on uptake and local integration	[35]
Generative AI	GPT-4-class LLMs, Gemini, Claude, RAG-enabled systems	Feasibility and bounded-task performance; very limited patient-centered outcomes	Early phase	Small samples, retrospective designs, hallucination risk	[44]

**Table 2:** AI implementation in infectious diseases.

Domain	Comparator	Key Metric or Outcome	Practical Take-Home Point
TB triage	Radiologist or standard triage pathway	Faster prioritization with strong discrimination	Useful where expert radiology capacity is limited
Sepsis detection	Vendor-reported or usual workflow performance	External performance may degrade substantially	Validation and threshold governance matter more than headline metrics
Host-response diagnostics	Standard adjudication or conventional diagnostic pathways	Earlier bacterial/viral differentiation and severity estimation	Useful as an adjunct when culture or definitive diagnostics are delayed
Stewardship support	Routine prescribing	Reduced mismatch and unnecessary fluoroquinolone exposure	Strongest when embedded in clinician-facing order workflows
Generative AI	Residents or specialists on vignettes/cases	Mixed accuracy and completeness; feasibility rather than effectiveness	Not yet ready for autonomous clinical use

**Table 3:** Economic impact of AI in infectious diseases.

Setting	AI Intervention	Comparator	Cost-Effectiveness	Study
Karachi, Pakistan	AI-based CXR TB triage	Standard smear/Xpert	Dominant: cost-saving. AI triage saved ~$4500–$12,600/1000 patients and averted ~13–15 DALYs (~$39–$40 per DALY).	[72]
Los Angeles, USA	AiCure video DOT for TB therapy	In-person DOT	Dominant: cost-saving. AiCure cost $2668 vs. $4894 and gave 1.05 vs. 1.03 QALYs (saving ~$2226 per patient).	[75]
ICU patients, Sweden	NAVOY^®^ Sepsis ML prediction	Standard ICU care	Dominant: cost-saving. Predicted sepsis 3 h earlier, saving ~€76/patient (via shorter ICU stays) and ~356 lives/year.	[76]
Ambulatory USA (EMR)	ML algorithm to detect undiagnosed HCV	Usual risk-based screening	Cost-effective: ICER ~$92,245/QALY (below $100 k threshold) at optimal operating point.	[77]
India (TB screening program)	AI-assisted CXR (qXR, Genki)	Standard CXR interpretation	qXR dominant: cost-saving (ICER ≈ −INR 9865 ≈ −$120 per case). Genki cost-effective: ICER ≈ INR 11,287 (≈$137) per case. Both ICERs are below India’s GDP per capita.	[73]

## Data Availability

No new data were created or analyzed in this study. Data sharing is not applicable to this article.

## Supplementary Materials

The following supporting information can be downloaded at: https://www.mdpi.com/article/10.3390/diagnostics16121827/s1, Table S1: AI applications in infectious diseases and their performance metrics; Table S2: Notable tools and regulatory status; Table S3: Representative AI studies and performance vs. standard care.

## Abbreviations

The following abbreviations are used in this manuscript:

AI	Artificial intelligence
AI-CDSS	AI clinical decision support system
AMR	Antimicrobial resistance
AMPs	antimicrobial peptides
AST	Antimicrobial susceptibility testing
AUC	Area under the curve
CAD	Computer-Aided Design
CE	Conformite Europeenne
CHEERS-AI	Consolidated health economic evaluation reporting standards for interventions that use AI
CNN	Convolutional neural network
CXR	Chest-X-ray
DALY	Disability-adjusted life year
DL	Deep learning
DOT	Directly observed therapy
ED	Emergency Department
EHR	Electronic health record
EMR	Electronic medical record
FHIR	Fast healthcare interoperability resources
GDPR	General Data Protection Regulation
HIPAA	Health insurance portability and accountability act
HIV	Human immunodeficiency virus
ICER	Incremental cost-effectiveness ratio
ICU	Intensive Care Units
ID	Infectious Disease
IVDR	In vitro diagnostic medical devices regulations
LLM	Large language models
MALDI-TOF	Matrix-Assisted Laser Desorption/Ionization—Time of Flight
MDR	Medical devices regulations
MDRO	Multidrug-resistant organisms
ML	Machine learning
NLP	Natural language processing
PPE	Positive predictive value
QALY	Quality-adjusted life year
RAG	Retrieval-augmented generation
TB	Tuberculosis
US	United States
WHO	World Health Organization
