# Supplementary material for: Artificial Intelligence in Infectious Disease Care: Selected Applications in Tuberculosis, Sepsis, and Antimicrobial Stewardship
Source: Diagnostics (Basel). 2026 Jun 12;16(12):1827. doi: 10.3390/diagnostics16121827 (PMC13297825; doi:10.3390/diagnostics16121827)
Supplement: Supplementary file 1 [file diagnostics-16-01827-s001.zip › diagnostics-4322948-supplementary.pdf]

Review

Artificial Intelligence in Infectious Disease Care: Selected Applications in Tuberculosis, Sepsis, and Antimicrobial Stewardship

Olga Adriana Caliman-Sturdza [1,2], Roxana Elena Gheorghita [1,*], Roxana Filip [1,2,*] and Andrei Lobiuc [1]

[1] Faculty of Medicine and Biological Sciences, Stefan cel Mare University of Suceava, 720229 Suceava, Romania; olga.caliman-sturdza@usm.ro (O.A.C.-S.); andrei.lobiuc@usm.ro (A.L.)
[2] "Sfântul Ioan cel Nou" Emergency Clinical Hospital, 720262 Suceava, Romania
* Correspondence: roxana.puscaselu@usm.ro (R.E.G.); roxana.filip@usm.ro (R.F.)

Supplementary Table S1. AI applications in infectious diseases and their performance metrics

Application	Representative system or study	Data and validation	Key performance metrics	Evidence maturity	References
TB diagnosis from immunologic/lab data	cforest ATB vs LTBI model	892 discovery, 263 validation; independent validation cohort	Test AUC 0.978; sensitivity 93.39%; specificity 91.18%. Validation AUC 0.963; sensitivity 92.80%; specificity 89.86%.	Retrospective diagnostic model with external validation	[23]
TB diagnosis from routine labs	GBM model	2,619 discovery, 942 validation across 2 hospitals	Test sensitivity 84.38%; specificity 92.71%. Validation sensitivity 87.63%; specificity 91.34%.	Retrospective external validation	[24]
Commercial TB CAD products	South Africa prevalence-survey comparison of 12 products	External validation in 774 participants	Lunit and Nexus AUCs near 0.9; some products maintained >90% sensitivity across broad thresholds; performance worsened in older adults, prior TB, and HIV	External validation of commercial tools	[25]
Population-scale TB screening	AIRIS-TB	Temporal test on >1.0 million CXRs plus external datasets	AUC 98.51%; overall FNR 1.57%; TB-FNR 0% after expert review correction; potential workload reduction up to 80%.	Large-scale temporal validation; workflow-oriented but not yet prospective trial	[26]
Sepsis triage from structured triage data	Gradient Boosting	Retrospective cohort of 189,617 ED triage presentations	AUC 0.83; sensitivity 0.74; specificity 0.78; PPV 0.77; NPV 0.74.	Large retrospective proof-of-concept	[30]
Deployed sepsis prediction in routine EHR	Epic Sepsis Model external validation	Independent hospital-wide external validation	AUC 0.63; sensitivity 33%; specificity 83%; PPV 12%; NPV 95% at threshold 6.	Real-world deployed model with disappointing independent validation	[8]
FDA-authorized sepsis AI	Sepsis ImmunoScore	Prospective multicenter development plus inter-	AUC 0.85 derivation, 0.80 internal validation, 0.81 external validation; external mortality rose from 0.0% in	Prospectively studied and FDA authorized	[45]

Application	Representative system or study	Data and validation	Key performance metrics	Evidence maturity	References
		nal and external validation across 5 US institutions	low-risk to 18.2% in very-high-risk groups.		
Host-response diagnostic/prognostic AI	TriVerity	1,441 adults across 22 EDs; clinical validation; FDA cleared	Bacterial AUROC 0.83; viral AUROC 0.91; severity AUROC 0.78; high-band specificity 90.7%, very-high-band specificity 95.5%.	Multicenter clinical validation and regulated product	[46]
Outpatient stewardship implementation	UTI Smart-Set	Real-world implementation across 171,010 UTI diagnoses	Recommendation acceptance 66.0%; mismatch 8.9% vs 14.2%; ciprofloxacin use 6.4% vs 32.9%.	Real-world implemented decision support	[35]
Bloodstream-infection prediction	Time-series LSTM	Retrospective single-center study with temporal hold-out	Hold-out AUROC 0.97 and AUPRC 0.65 for LSTM vs AUROC 0.74 and AUPRC 0.48 for static models.	Temporally validated retrospective model	[48]
Antibiotic discovery	AMPSphere	63,410 metagenomes and 87,920 genomes; preclinical validation	863,498 peptides identified; 79/100 synthesized peptides active *in vitro*.	Preclinical discovery platform, not clinical implementation	[37]

Supplementary Table S2. Notable tools and regulatory status

Tool / product (focus)	Organization	Primary clinical use	AI method (as described in public sources)	Reported performance metrics (examples)	Regulatory status (publicly documented)	References
Sepsis ImmunoScore (sepsis risk stratification within 24h)	Prenosis	Risk stratification for suspected sepsis in ED/inpatients, used with clinical assessment	Locked AI/ML algorithm using up to 22 predetermined EHR inputs	Public excerpts emphasize 4 risk categories; specific sensitivity/specificity values are not extracted from the limited excerpted text here	US De Novo authorization (DEN230036; decision date 2024-04-02)	[45]
TriVerity Test System (bacterial vs viral vs noninfectious + severity)	Inflammatix	ED triage for suspected acute infection/sepsis; supports antimicrobial decisions and escalation	Host-response gene expression classifier (29-mRNA RT-LAMP system)	Indicated to aid differentiation and 7-day severity needs; specific diagnostic/prognostic metrics appear in clinical studies and authorization documents but are not fully enumerated in the excerpted lines shown here	US 510(k) clearance (K241676; decision date 2025-01-10)	[57]
NAVOYCDS (sepsis detection decision support)	AlgoDx	Decision support for sepsis detection using monitored patient data	Proprietary algorithm (sepsis detection CDS)	Performance metrics not specified in the excerpted indication statement	US 510(k) clearance (K240558; decision date 2024-07-22)	[58]

INSIGHT CXR Triage (radiology triage tool relevant for infection-related findings)	Lunit	Chest radiograph triage/prioritization (supports faster review for critical findings)	ML-based radiology prioritization software	Public listing does not include performance metrics; see submission summary in the device database	US 510(k) clearance (K211733)	[59]
qXR (TB-related abnormality detection on CXR)	Qure.ai	CXR interpretation assistance for TB-related abnormalities, enabling downstream testing/triage	AI-based CXR interpretation	Literature describes CE marking and clinical workflow use; specific metrics depend on cohort/task	CE marking reported in implementation literature; additional pediatric clearance claims exist but are not independently verified here beyond cited sources	[60]

Supplementary Table S3. Representative AI studies and performance *vs.* standard care

Study (short label)	Population / setting	AI method	Comparator	Key performance metrics reported	Time-to-diagnosis / time-to-action implications	Regulatory status note	References
TB CXR triage head-to-head evaluation	23,954 chest X-rays; high TB-burden triage setting	Five commercial DL algorithms	Radiologist readings; WHO triage TPP thresholds	AUC ~0.85–0.91; algorithms outperformed radiologists	Supports rapid triage to molecular testing; primary value is prioritization	Commercial products; CE status varies and is not fully enumerated here	[22]
External validation of a widely implemented sepsis model	38,455 hospitalizations; external test site	Proprietary sepsis risk model	Usual care alerting and/or internal vendor-reported performance	Hospitalization-level AUC 0.63; sensitivity 33%; specificity 83%; PPV 12% (reported in editorial summary)	Demonstrates risk of poor generalization; can increase false alerts and dilute actionability	Not described as a regulated marketed device in the cited study	[8]
Real-world sepsis DL integration	Routine care deployment site (quality improvement)	DL sepsis detection + management platform	Pre-implementation workflows	Focus is integration, not headline AUC; documents operational barriers and implementation structure	Emphasizes clinical “last-mile” integration and alert routing for earlier recognition	Institutional deployment; regulatory status not specified	[53]

29-mRNA host-response classifier validation	688 ED patients with suspected infection	Transcriptomic classifier (bacterial/viral) + severity model	Forced/consensus adjudication; outcomes	AUC 0.76 (bacterial), 0.89 (viral), 0.77 (30-day mortality)	Enables earlier etiologic guidance prior to definitive cultures and supports early escalation decisions	Study-level evidence; product regulatory status depends on specific commercial instantiation	[68]
MALDI-TOF AMR prediction	Clinical isolates with linked susceptibility phenotypes	ML prediction from MALDI-TOF spectra	Culture-based AST workflow	Framed as direct AMR prediction; notes culture-based methods can take up to ~72h	Potential to deliver actionable resistance signals earlier in the diagnostic timeline	Research evidence; clinical deployment status varies by site/product	[28]
AI-CDSS resistance prediction RCT	1,600 S. maltophilia infections; 800 vs 800	AI-CDSS using MALDI-TOF-informed prediction	Standard care without prediction	14-day mortality 11.5% vs 15.1%; improved confidence and appropriate antibiotic selection	Explicitly claims “1 day earlier” resistance prediction, affecting early therapy adjustment	Trial evaluated a CDSS; regulated status not specified in the publication abstract	[34]
Outpatient UTI order-set decision support	Large outpatient organization; large case volume	ML resistance forecasts + guidelines in an order set	Routine prescribing patterns	Reported reductions in mismatch and ciprofloxacin use; example figures include 8.9% vs 14.2% mismatch (when followed) and 6.4% vs 32.9% ciprofloxacin	Can reduce inappropriate antibiotic exposure at point-of-prescribing	Operational tool; regulated status not specified	[35]

*Notes: “Performance” metrics summarize what is explicitly reported in the cited source abstracts/snippets; values can differ by threshold, subgroup, and reference standard. “Time-to-diagnosis” gains are often inferred from workflow changes (e.g., earlier triage) rather than measured as randomized time endpoints unless explicitly stated.
